# Law Department of the University of Louisville

**Published:** 1847-09

**Authors:** 


### LAW DEPARTMENT OF THE UNIVERSITY OF LOUISVILLE.

Prof. Duncan having been called by a portion of the citizens of Kentucky to represent them in the Congress of the United States, has resigned his chair in the University of Louisville. We are glad to learn that the Hon. Ephraim M. Ewing, a gentleman every way worthy to be his successor, has been called to the vacancy by the board of trustees. Mr. Ewing was for many years a judge of the Court of Appeals, and in May last resigned the office of Chief Justice of Kentucky. He is still in the prime of intellectual life, and brings with him into the University a reputation for uprightness and purity, professional learning, and mental vigor, which will give additional character to the department in which he is to labor. As a jurist he ranks among the ablest in the United States, and as a speaker he is forcible and eloquent. We congratulate the friends of the University on this excellent appointment, and take great pleasure in stating that its Law department has the promise of as prosperous a career as that enjoyed by the department of medicine.

